# Evaluation of a participatory action project to improve safety and outcomes in maternity care

**DOI:** 10.1186/s12961-025-01319-7

**Published:** 2025-04-24

**Authors:** Marie-Clare Balaam, Gill Thomson

**Affiliations:** 1 Research in Childbirth and Health (ReaCH Unit), School of Nursing and Midwifery, University of Central Lancashire, Preston, United Kingdom; 2 Parental and Infant Nutrition and Nurture Research Team (MAINN), School of Nursing and Midwifery, University of Central Lancashire, Preston, United Kingdom

**Keywords:** Participatory action, Co-production, Collaboration, Safety, Maternity care, Maternal violence, Perinatal, Ethnic minorities

## Abstract

**Background:**

Maternal violence, in terms of obstetric violence and/or the disrespect and abuse of women and birthing people accessing maternity care, is a global concern. This mistreatment and experience of maternal violence and harm has negative physical and psychological impacts on women, birthing people and their babies. This paper evaluates a multipartner project which aimed to co-produce specialist resources to support women and birthing people who had experienced violence and harm. The evaluation sought to understand the collaborative and co-production processes employed and to identify recommendations and learning from the project.

**Methods:**

An ethnographic-based evaluation based on action research and participatory action research principles was undertaken using qualitative interviews, documentary review and observations. The data were analysed using reflexive thematic analysis.

**Results:**

A total of 18 interviews were conducted with 21 participants from the lead, project partner and onward grant recipient organizations. In addition, 80 documents were reviewed, and 9 collaborative group meetings and 2 in-person events were observed. Factors which supported and inhibited effective collaborative working and co-production were identified in five aspects: ensuring inclusivity, clarity and transparency, building and maintaining relationships, collaboration and cooperation and active learning.

**Conclusions:**

Effective collaborative co-production needs to consider issues of inclusivity and diversity and to ensure clarity and transparency in terms of remit, commitments and finances. Building and maintaining relationships between partners and communities by creating a safe space for participation and inclusive leadership was crucial. Recommendations from the evaluation include the need to ensure mechanisms for clear communication within projects from their inception as well as the need to acknowledge and proactively address issues of diversity and inclusivity throughout all aspects of the co-production process to support the fullest participation from diverse stakeholders.

## Background

Maternal violence, here defined as including, obstetric violence, female genital mutilation or cutting (FGM/FGC), gender-based violence and domestic abuse and/or the disrespect and abuse of women and birthing people accessing maternity care, is an issue of global concern [1, 2]. This mistreatment, which includes physical or verbal abuse, humiliation, lack of confidentiality, lack of fully informed consent, withholding pain relief and infringement of privacy, represents a violation of human rights and can impede women’s and birthing people’s autonomy, individual agency and control over their bodies [3, 4]. The experience of maternal violence and harm has negative physical and psychological impacts on women and their babies, including post-traumatic stress disorder symptoms, difficulties bonding with the baby, breastfeeding cessation and negative impacts on future reproductive choices [5–8]. Research also suggests that some women and birthing people are at higher risk of harm and violence during maternity care due to a range of intersectional factors, including ethnicity, mental illness, socioeconomic status, histories of trauma and abuse, marital status, religion, caste, class, language, parity, religion and age [9–11], and are less likely to seek out help and support [12, 13].

This paper reports on an ethnographic-based evaluation of a project undertaken to address some of the issues relating to maternal violence, as identified above, through the co-production of specialist information and resources to be hosted on a popular UK-based parenting app. The project was underpinned by a commitment to co-production, here defined as “engaging stakeholders in the implementation of previously set solutions to an already agreed problem, in prioritizing the optimal usage of available resources” [14] and sought to use principles of co-production throughout the project to co-develop this specialist content, detailed below. It was also underpinned by a participatory action research (PAR) framework [15, 16] where PAR is defined as the “systematic collection and analysis of data for the purpose of taking action and making change” by generating practical knowledge [17].

The evaluation was commissioned by the lead organizations and undertaken by two experienced maternal health researchers. The researchers were directed to work with the lead organizations to develop an evaluation protocol to explore the ways in the PAR approach was undertaken in the context of this work. This focus reflects the increasing value placed upon using approaches such as PAR and co-production when seeking to bring about improvements in maternity care and women’s health in a way in which the experiences and perspectives of those effected are included [18, 19]. The aim of the paper was to explore the learning from this project which sought to use these approaches in a challenging and sensitive area of maternity care with a large group of collaborators. To this end, the evaluation sought to understand the collaboration processes, how complex relationships were navigated, how sensitive and representative service-user driven content was co-produced and to identify learning and recommendations for future projects, as well as for transferable learning.

The project, which took place between late 2021 and March 2023, was led by two established third sector organizations working in the parental wellbeing and advocacy space and involved collaboration (and financial remuneration) with 17 community and third-sector organizations representing communities from across the United Kingdom as project partners. Different levels of remuneration were provided across the different partner organizations. The project took an intersectional approach by working with stakeholders from diverse communities, including groups representing ethnic minoritized communities, those facing socio-economic marginalization, young parents, those experiencing gender-based violence and domestic violence and other underrepresented and seldom heard communities. This approach was designed to capture the complexity of experiences of women and families and to produce content that was representative of those who were most likely to experience violence and harm. It involved working with these project partners to co-develop the specialist content using a range of strategies including individual consultations, in person co-production events, regular monthly whole project online meetings, small group meetings, one-on-one meetings, training and two hybrid conference events.

Both written and filmed evidence-based, culturally sensitive content was designed to guide women and birthing people through safe maternity journeys to ensure cultural safety and to encourage appropriate help-seeking. It also included content for minoritized parents to enable them to act as agents in their maternal experience, and for fathers and male partners to proactively increase the use of resources that educate, inform and signpost relevant care services for women and birthing people. Other resources included a podcast series and training materials for healthcare professionals. The co-production of the material was undertaken through the content production team from one of the lead organizations, holding a series of meetings, including one-on-one meetings with all delivery partners. The purpose of these meetings was to suggest content for the resources, provide feedback on the proposed content and to review the final versions. Methods of working were flexible and evolved over the project as a response to conversations with delivery partners, for example, initially several subgroups had been envisaged, but in the end only one subgroup was used. Additionally, two in-person events were held to co-develop content and work on strategies to maximize the sharing and impact of the resources. Training was provided for all delivery partners on the use of the app and to support the promotion of the app and the new content within their communities. The project additionally provided eight grants to organizations with expertise in working with obstetric violence, domestic abuse/violence and FGM/FGC across England, Scotland and Wales to support them to develop a discrete service or practice that aimed to champion women and girl’s self-advocacy in relation to safety from violence and harm.

## Methods

### Research design

In line with the PAR approach adopted by the project, a theoretical framework based on action research methodology [20] and PAR “good practice” principles [21] was developed to guide data collection (providing the basis for interview schedules, observations and documentary analysis), analysis and interpretation. The theoretical framework was employed to understand how participatory and inclusive approaches to collaboration and co-production were applied within practice and the ways in which this was experienced by those involved. It included considerations of integrity in terms of how participants could engage in the project in an authentic manner, equality and inclusion, mutual respect, opportunities for collective action, action learning and evaluating how the project had made a difference to participants.

### Data collection

Three different forms of data collection were used for the evaluation, outlined as follows:

- Online interviews:
  Qualitative semi-structured online interviews were undertaken by the researchers with staff from the lead, partner and onward grantee organizations. All individuals were emailed an information sheet and consent form and asked to contact the evaluation team if they wished to take part. Issues of possible disparities in power were acknowledged and various attempts to mitigate these were used. This included researchers meeting participants (i.e. in the online meetings, or co-production events) prior to the interview and reassurance that participation was voluntary. All participants had significant roles within with organizations and were experienced at advocacy and speaking about their work and experience.
  Consent was recorded verbally before the interview began and stored separately from the interview recording. Interview questions were based on the theoretical framework and explored roles within the project, relationships between partners, how information was communicated, how outputs were produced, how decision-making was agreed upon, how any disagreements or conflicts were managed, opportunities for learning and any recommendations for future projects.
- Documentary analysis:
  Project documents were analysed, data extracted and mapped against the theoretical framework. Documents included the initial grant application, planning documents, email communications, evaluations of events, project updates and notes from whole team meetings.
- Observation:
  Observations of meetings and events including project planning and content development events were undertaken by research staff. Contracts between the lead organizations and project partners provided approval for researchers to observe monthly project partner meetings either live or to watch previously recorded meetings. These observations were used by the researchers to add depth to the understanding of how the project was operationalized, rather than to document individual responses, and data from these observations are not shared in this paper.

### Data analysis

This study used methodological triangulation [22] using different methods to study the same phenomenon. Interview data were transcribed and these transcripts, along with extracts from documents, were uploaded to MAXQDA (a qualitative data software programme). A deductive reflexive thematic analysis [23] approach was used to analyse the data. This involved in-depth reading of the interview transcripts and documents as a whole dataset to ensure familiarization with the data followed by line-by-line coding. All data were then synthesized into subthemes and themes under predefined broad concepts from the overarching theoretical framework. Data analysis was undertaken by both authors and was an iterative process in which themes were developed, reviewed and refined until consensus was reached. The final interpretations were shared at an event at which all partners were invited, additionally the final report was shared with the lead partners who had the opportunity to review and comment on the findings.

### Ethics

The research was carried out following the University of Central Lancashire’s Code of Conduct for Research and an Ethical Principals Framework and in accordance with the Declaration of Helsinki. Ethics approval was obtained from the University of Central Lancashire, following review by the Health Ethics Review Panel (unique reference no. HEALTH 0352).

## Results

A total of 18 interviews were conducted with 21 participants (3 interviews had 2 participants), and all interviewees were drawn from third-sector organizations which work with women, partners and families within a maternity and parenting setting and included groups specializing in supporting minoritized and marginalized communities. The participants included seven staff from the two lead organizations, one representative from eight of the project partner organizations and six staff from onward grant recipient organizations. Participants represented organizations from England, Scotland and Wales. In total, 11 participants were from minoritized ethnic groups and 19 were female and 2 were male, and are represented as partner organizations (PO), lead organizations (LO) and onward grant recipients (OGR) in the sections below. Interviews were undertaken between November 2022 and March 2023. A total of 80 documents were reviewed, and 9 collaborative group meetings and 2 in-person events were observed. Key findings in relation to factors which supported and inhibited effective collaborative working and co-production are presented in five themes below: ensuring inclusivity, clarity and transparency, building and maintaining relationships, collaboration and cooperation and active learning. The recommendations made to improve effective collaboration and co-production are included in the discussion of the findings, contextualized by the wider literature.

### Ensuring inclusivity

This theme explores the ways in which the project sought to ensure inclusivity within the development of the outputs as well as in the management of the project.

While diversity and inclusivity were a key underpinning ethos of the project, participants highlighted a notable lack of diversity within the leadership of the project. One participant identified this contradiction as problematic:

I haven’t seen anyone in that role, or anyone in those high roles that is from that diverse background … it’s not the best if you’re going to be talking about representing diversity and inclusion, you need those people up at the top, so that you know that it’s being done right (Participant_07_PO).

Some also viewed this lack of diversity and representation within the leadership as potentially reproducing non-inclusive practices and unequal power relations:

There should have been more of a conversation around full partnership .... [otherwise] it sounds like minions, to do the groundwork, which is basically the same power dynamics that we see across every single majority of establishments that we know, is based on institutional structural racism … It does seem like OK, this is a great opportunity, but when you look at it properly, it actually does seem like we’re going to still hold on to the power, and it kind of defeats the whole purpose of this kind of work (Participant_03_PO).

Despite this lack of diversity in leadership roles, many of the project partners were from minority ethnic backgrounds and represented organizations who worked with people from different communities including minoritized women, fathers, women experiencing gender based or domestic violence. One participant reflected that:

I think that the delivery partners are quite diverse. You know, it was nice to be in a group of people where white faces weren’t the majority, you know, and I thought that was noticeable, which was good (Participant_05_PO).

This commitment to diversity was felt to go beyond that of providing a space for community voices to be heard by providing opportunities to “frame the project” and to be directly represented in the content produced:

The fact that my community will see themselves represented in that, again, is another opportunity for us to support them, and raise them into a space where, actually you belong here. You don’t have to fit in here. You actually belong. This is a space for you too (Participant_06_PO).

Others noted how being provided with financial remuneration for their involvement helped to demonstrate the project’s commitment to working with diverse communities:

The fact that someone like me, who has an organization that works with those people has been engaged, and financially remunerated for that engagement, and the respect for my knowledge I genuinely think that that is a start, and a step into the right direction of supporting the community. People like me cannot continue our work if we don’t have money, and we don’t have opportunities (Participant_06_PO).

While the need to be inclusive and ensure representation was at the heart of the project, it was recognized that having too many partners could reduce the commitment and sense of personal responsibility of those involved in that:

I know if I don’t attend the meeting, I’m sure that at least 16 or 15 others would be there, so that responsibility has been, kind of, removed and nobody I guess is taking full responsibility, or feeling that the onus is purely on them. But if there was four or five partners, my presence would absolutely be needed in every single session, and I would understand that (Participant_03_PO).

It was also felt that while trying to ensure a range of experiences, care was needed to not overrepresent particular voices, whereby including multiple partners with the same area of focus could lead to individuals feeling there was a sense of doing “inclusion for inclusion’s sake” or that their views were not adding value:

If everyone’s echoing the same thing, and it’s like literally 10 partners, 10 Black maternal health partners involved, all trying to do the same thing … I feel like I don’t need to be involved at all (Participant_03_PO).

Several partners commented on how the relationships with communities involved should be maintained beyond the programme, both in terms of funding and a wider sense of ongoing commitment to working with these groups:

There has been a quite a bit of data extraction from really unique organizations, it is therefore important to really support our organizations around funding and development. I believe the partnership should continue in some way or form and should not just close it down now it has achieved its objectives (Document 01: Delivery partner action plan).

### Relationship building

In this theme we report on insights that reflect the importance of building and maintaining positive, respectful and productive relationships to support effective collaboration and co-production.

At the start of the project an initial Zoom meeting with all project partners was held to help establish relationships, followed by regular monthly online project partner meetings. Some participants found these helpful and noted that:

If we hadn’t had those calls to be able to get to know each other’s personalities, to hear more about their organization, it would have felt more disjointed …that really helped to give that personal touch to the whole thing (Participant_10_LO).

Most meetings took place online. While for some this had worked well, allowing them to balance their daily commitments with this work, others felt the opportunities for in-person contacts were important for deepening relationships:

I did like the mix of in person and online. I think that worked best. All in person wouldn’t work for me, because I wouldn’t be able to go. All online would work, but I don’t think it would have been as cohesive. I think those days where we met up really gave it a pulling together and getting to see people that you’ve only spoken to online (Participant_05_PO).

Other participants suggested that ideally “people need to know each other and there needs to be a level of trust already” (Participant_09_PO) for collaborative work to be done and that this had been challenging on Zoom. It was felt that it may have been beneficial to have had more time to build trust and relationships before the start of the co-production work, particularly when working in such a sensitive area.

Participants also reflected on how the nature of the project had allowed the creation of relationships and connections which would potentially reach beyond the project for long-term positive impact:

Once that [project] does finish, it doesn’t mean we should come to a standstill. We should keep these relationships, right? …that’s what’s worked very well is that you’re able to connect (Participant_04_PO).

The relationships created provided a sense of connection with others working within the area of maternal violence and were a source of mutual support for people working in what was a challenging field “because it can feel very isolating …like you’re fighting against these big systems” (Participant_17_OGR). One participant noted that these connections made them feel “less alone and isolated as an outspoken voice” (Document 01: Delivery partner action plan).

### Clarity and transparency

In this theme, we report findings relating to the need for clarity and transparency to ensure that there were clear expectations of the roles and involvement for all project partners.

Participants from both lead organizations recognized that initially the vision for the project had not always been adequately communicated and that there had been a lack of clarity around project management processes, both of which impacted on aspects of project delivery. Moreover, while one project partner felt an initial email had provided them with a clear understanding of what was expected of them, others lacked this sense of clarity, feeling that there was some “confusion” and “ambiguity”. One noted “I’m not actually sure why I’m there” (Participant_09_PO) and another felt they “didn’t really have a clear definable goal when we first started” (Participant_10_PO). For some partners these feelings resolved as the project progressed; one noted how “at the beginning, it wasn’t made very clear” but over time “I was able to slowly see the vision on paper, and then it was brought to life” (Participant_04_PO).

For some partners a level of ambiguity seemed acceptable in a participatory and collaborative project, with one stating “I always knew this was a bit of an experimentation” (Participant_07_PO). However, others found the lack of clarity more challenging:

Do I feel like I have clarity of understanding of what’s going on? Do I have clarity of what’s expected of me? Do I believe in you as an organization? Like do I believe in your ability to deliver this? Do I feel like there’s been transparency around everything? And I was like all those things it’s a no (Participant_09_PO).

Participants highlighted a need for “more clarity from the beginning of what the goals are” and that this lack of initial lucidity had created time pressures “of trying to get everything done” (Participant_10_LO). Another felt that the confusion was compounded by the numbers of partners involved, stating:

Figuring it out as you go along with so many partners is just not good … I think you need to need to know exactly what you’re trying to do before you start (Participant_09_PO).

Some project partners felt that they would have been more able to effectively plan their time, balance other commitments and have a clearer idea of the level of input expected if the expectations of their involvement had been identified more explicitly at the start. It was suggested that there needed to have been:

Really clear and transparent contracts – you are required to attend this many delivery partner meetings to do XYZ for the conferences; you’ll be paid on the delivery of these things… there should be accountability on both sides (Participant_11_LO).

Lead staff became aware of this lack of clarity, with one document noting, “we realize that there are some questions around what being a delivery partner means” (Document 02: Where we are at: June 22). To address this, additional information was provided in several ways including discussions in project partner meetings, one-on-one meetings and in email communication and shared documents.

A further area of concern related to a lack of transparency around finance within the project. Project partners wanted to understand both how the money was going to be used, “what are you doing with this money?” (Participant_06_PO), as well as how the money had been allocated amongst the project partners:

I think the financial clarity should be there all the time anyway just to let people know that there is no need for you to worry, because everything is allocated nicely and.... I think that gives that transparency. It gives everybody kind of that feeling of, yeah, I get what’s going on (Participant_06_PO).

During the project some partner organizations became aware of a disparity in the payments made to the different partners. Those who were aware of this expressed concerns over this lack of equity. One noted that:

We’re all delivery partners, so we’re all meant to be wearing the same hat. And so, if you’ve asked one group that does basically identical things to another group, and they’ve been given additional funding, or additional roles, then it might be like, oh, what about me ... wondering why one might have gotten less or more responsibilities, or more budget, or more attention than the others (Participant_03_PO).

Another suggested that this situation could have caused tensions and damaged relationships between the lead and project partners:

The key thing is that people talk. It’s a community where, relationships have been formed, trust has been built, and people talk. So, we are all being paid to be a delivery partner, but it quickly came to light that not everyone has been given the same amount (Participant_04_PO).

Overall, the inequity and confusion around funding was believed to be an unnecessary situation that could have been easily remedied:

We don’t need to know the exact amount, but at least understand, or know the rationale, to why that has happened, and then that can put that issue to bed (Participant_04_PO).

### Collaboration and cooperation

Participants reflected on how collaborating with a large group of individuals with different views, and approaches necessitated a respectful and emotionally safe approach, particularly with the sensitive and personal nature of the project. Overall, project partners generally felt they had directly influenced content production and that integrating the “voices of service users” meant that “authentic” (Participant_03_PO) material had been co-produced, and that protected against further marginalization of those communities.

Project partners also felt that the inclusion of so many voices within the content and in the process of content production had added value in terms of the potential impact of the resources in terms of broadening the “audience that we’re reaching, and, you know, more change, and it is more impactful” (Participant_04_PO). However, the process of collaboration and cooperation could be challenging, as it involved negotiating differences between the lead organizations as well as between the wider project group. It was noted that at times the different approaches of the two lead organizations, one characterized as being “more activist and opinionated” and the other as feeling “the need to be very neutral” was described as “quite challenging” (Participant_11_LO). One participant felt that despite the number of organizations involved, there had not been “any clashes, because we’re all on the same page” (Participant_04_PO), suggesting that a unifying goal overrode differences. However, a more representative view was that different perspectives were acknowledged and that these were generally managed in a sensitive way by people being “respectful of other people’s viewpoints” (Participant_05_PO).

Participants referred to how the project leads had created a safe space in which differences could be explored and used productively. This space was facilitated by training on trauma-informed practice and the use of these principles throughout the project. Partners were warned about issues which may be particularly challenging (such as issues around FGM/FGC), and opportunities were provided to talk about any issues of concern, including access to a free debriefing service provided by a clinical psychologist. One participant noted that the project leads had been:

Mindful of the subject matter, of people’s different views, checking in with people, knowing that people could be triggered by things. I think that side of things has been managed really, really well. It has been a safe space where people can talk and express their views and hopefully feel comfortable that that no one’s gonna be judged (Participant_07_PO).

The creation of a safe environment facilitated a sense of trust, respect and support, with partners speaking about having been “listened to”, “heard”, “respected” and “understood” within the project. They felt that they were “valued” due to their “personal knowledge and experience” and that they were seen as “the experts in our provision” and in the communities where they worked. One participant reflected:

I felt valued, you know, in bringing the opinions that I’ve brought, that maybe was slightly different from some other people, on behalf of the mums that I worked with (Participant_18_OGR).

Furthermore, due to the potential for unequal power dynamics between the lead organizations and delivery partners, project leaders emphasized adopting an inclusive leadership style to promote equality and engagement:

I have tried to instil the sense of leadership by being quite quiet in delivery partner meetings. I’ve never felt like I’ve been a facilitator in those. I feel like they’ve been, sort of, town hall style. Everybody contributed, everybody led (Participant_01_LO).

And in addition, to ensure that there were opportunities for all partners to be included:

I literally said, we need to have an event that is around how we would share [the resources]. Who wants to run it? And [name] put a hand up, and on that day, it was absolutely [name] running and leading that. I was a participant on that day. That is how I would have liked more of the programme to be (Participant_01_LO).

Some of the project partners reflected on how this approach mirrored the underlying principles of the project:

[the project lead] really mirrored what the project is trying to do around, you know, giving women, families, self-advocacy, self-agency, in this space (Participant_02_PO).

The vision of the project, in terms of content creation, was that it would all be co-created with project partners and that minoritized voices were to be the “driving voices” within the resource creation: “we want the voice of black, brown, South Asian people to be dominant within the new resource creation” (Participant_01_LO). It was therefore imperative to establish what co-production meant in the context of the project, with this topic discussed at the initial project partner meeting as a way of starting this discussion/process:

I think exploring together, and actually your very first delivery partner meeting being a case of, we’ve got this lump sum of money for delivery partners, what is co-production? How do we define that? How do we work to work out who’s gonna do what, is actually deeper co-production, than going, you’re all gonna get this amount of money, you’ve all signed a contract, now off we go, let’s co-produce together (Participant_01_LO).

However, the idea of co-production within the project was not without challenges. For instance, in consideration of the need to ensure emotional and psychological safety for all involved, concerns were expressed about whether there had been sufficient preparation to ensure the partners' “readiness for exposure to these conversations” (Participant_01_LO). The use of an evolving style of co-production that developed with the project, as opposed to a more structured one, was used. However, it was acknowledged by one staff member that this approach had been in this context a challenging approach:

Co-production is all well and good, but needs some container, and we didn’t build the container well enough, … I’ve probably been overly concerned that I’m restricting voices, whereas actually what I now recognize is people needed greater steer you can still lead with boundaries in a co-production methodology (Participant_01_LO).

And one project partner suggested that a more formalized or boundaried approach could have helped partners to know “Why they’re there, what we’re trying to achieve, what they’re bringing to the table, what’s expected of them” (Participant_09_PO).

### Active learning

This theme explores the ways in which the project facilitated active learning at individual and organizational levels.

The opportunity for diverse partners to interact within a safe space facilitated personal reflection and learning. Whilst one participant felt they had not “learnt much to be honest” (Participant_09_PO), most described experiential learning as they shared and developed knowledge, gaining new perspectives on their own work through hearing from others who had different ideas. Participants detailed how they had gained “a new level of understanding” (Participant_05_PO) and had a “really fresh perspective of some of the issues” (Participant_12_LO) which were outside of their usual areas of expertise. Some considered that their involvement had provided knowledge of other specialist organizations and services that complemented their own, enabling them to signpost their clients to other services and to offer wider benefits to those they support.

The space created within the project provided a unique and “rich” opportunity for partners to explore issues relating to maternal violence. One commented how:

I really don’t feel like there is enough space for that anywhere else within maternity, to actually explore what’s broken, what needs fixing (Participant_01_LO).

Participants repeatedly highlighted how the creation of a safe space in which all partners could work and within which differences could be used productively, was a crucial aspect of the project. They noted that they felt able to “have those conversations around where we see things, and then come together” (Participant _02_PO) and that people were “respectful of other people’s viewpoints” (Participant_05_P0) and that:

It has been a safe space where people can talk and express their views and hopefully feel comfortable that that no one’s gonna be judged, or no one’s gonna be thought wrong of for anything they may say or think (Participant_07_PO).

This opportunity enabled a deepening of understanding and appreciation of intersectional issues that were at the heart of the project, and the complex ways in which different groups and individuals may be affected by maternal violence and wider issues of inequity:

My own learning is that there are so many layers to experiences … There are so many people that have different things that they need to feel safe from. And so, when we look at all those different people, it’s not a matter of who's is worse than the other. It’s a matter of what is available to that person when they feel unsafe (Participant_06_PO).

While these conversations could be challenging, they were managed in such a way as to make them a valuable learning opportunity for those involved. One participant reflected:

There was some things that people did find challenging, but then they would come back …. [and] say like, oh, when so and so said that in the last call I found that really challenging or it even triggered me … but now I can see why they said that or I can identify with them or empathize with them more ….my impression was that it was a positive overall (Participant_10_LO).

Participants from lead organizations reflected on organizational-related learning, including how to undertake partnership working with partners who have different visions, and how to work safely within a contested and challenging space to ensure all participants remain safe and how to manage the unexpected “where it didn't go quite to plan” (Participant_10_LO).

## Discussion

This evaluation provides insights into key elements which support collaborative co-production. These include working to ensure inclusivity and diversity, the need for clarity and transparency, ensuring there are effective mechanisms for building and maintaining relationships with individuals and communities, creating the conditions to allow safe and positive collaborative working and making space for active learning.

Working with diverse and minoritized communities was prioritized in the project and valued by those involved. Recommendations on how to increase inclusivity and create further connections with communities were highlighted by partners. It was suggested that care should be taken to ensure that there was diversity throughout the project at all levels, including leadership roles, to prevent hierarchical power relationships. The project, while working with ethnically minoritized communities, lacked significant ethnic diversity within the leadership, although several members within the lead organization were from minoritized ethnic populations. The lack of ethnic diversity within the leadership and within the research team, with both researchers being white, means there was an increased chance of a misalignment of priorities, a lack of trust and a perpetuation of existing stereotypes and social hierarchies. The potential for power imbalances between larger, often more established and better-funded organizations, and smaller grass roots/community-based organizations, was also identified. This issue of the need to acknowledge and address power differentials which can often be present in co-production settings is discussed within the wider literature on co-production [24–28]. This literature suggests that the potential power differentials, which commonly replicate wider social hierarchies in terms of, for example, the positions of minoritized groups, or service users within professionalized settings, need to be openly acknowledged and efforts made to address them. One suggestion to address this issue is to ensure that all partners are acknowledged as bringing unique assets to the project and that these assets are all regarded as having equal value. In this way no knowledges, experiences or statuses are valued over others which facilitates a situation in which all partners are involved in decision-making and meaningful aspects of the project in a less hierarchical way [29, 30]. Other work has suggested that appropriate funding, training and reflection can all be helpful ways in which to acknowledge and challenge the replication of existing hierarchies [25].

It was suggested that the power asymmetry between different organizations could lead to a sense that the expertise and knowledge of smaller community-based organizations, which often represented minoritized communities, was being used to benefit larger organizations. This echoes benefit sharing concerns of preventing the exploitation of local knowledge and the need for fair distribution of the benefits and burdens arising from research [31]. It is therefore important that the input of these community organizations, in terms of specialist knowledge and access to service users, is appropriately acknowledged and valued and that there is reciprocal benefit to the community organizations involved [24, 25, 29]. Partnership work such as that undertaken in the this project can provide a range of benefits to the organizations involved and the communities they work with, including developing networks, future funding opportunities, learning opportunities and capacity building, as well as opportunities for service user involvement. The need to, where possible, provide a sense of continuity or to sustain links with groups beyond the limited duration of the project was also identified as an important aspect of the relationships between the larger and smaller community-based organizations and as a way of ensuring that communities did not feel undervalued or exploited.

In our study, clarity and transparency throughout all aspects of the project were identified as being important to ensure successful co-production. This included clarity around the aims of the project and the way in which the project would work, including what was meant by co-production, and the roles and responsibilities of partners. Other research has noted that, as identified in our project, there are different ideas of what co-production is and how it works [24, 28], and thus it is important to establish what is meant by this term within any project and to ensure that this is clearly understood by all involved [24, 32]. The literature has also identified the need for effective communication about all aspects of a project, including the purpose of the project, how it will be managed in practical ways including finance and feedback on how the project is progressing to ensure clarity for participants [25, 27, 33, 34].

The need to build and maintain positive and constructive relationships was found to be crucial for effective co-production, as reflected in wider literature [15, 25, 27, 35]. It was also suggested that it would have been useful to begin relationship building before the project formally began to facilitate feelings of connection and trust. Overall, different strategies were used to develop and maintain relationships in the project, such as in-person and online meetings and group sessions and one-on-one meetings. Despite the move to remote working, which increased throughout the recent coronavirus (COVID-19) pandemic, participants also valued opportunities to meet individuals in person [36]. This echoes other research that emphasizes that face-to-face meetings facilitate certain psychological interactions that are challenging to replicate in virtual meetings [37].

The project demonstrated some important insights into the ways of undertaking co-production with a large group of people with diverse views and when dealing with challenging subjects. Ensuring the inclusive and appropriate representation of disparate views, including the voices of those who are less heard, was a crucial aspect of this project. However, the management of this diversity, while often a key aspect of co-production work, can also be a challenging aspect of this methodology [24]. A mechanism by which the diverse views, experiences and perspectives were explored in a productive and respectful way was through the creation of a safe space. This safe space was enabled using trauma-informed practise, the promotion of self-care through free access to psychological support and through modelling good practice via inclusive leadership. The creation of a safe space has been identified within wider research as being a vital aspect of successful co-production. A safe space provides a location in which all participants are able and encouraged to express different views and perspectives as well as explore any tensions in a productive way [25, 35, 38, 39].

### Strengths and limitations

The strengths of this work are that it included different forms of data collection to provide an immersive perspective on how the project was undertaken. The use of a theoretical framework to guide data collection and analysis helped identify key issues that underpin a participatory co-production approach, although arguably the use of a deductive approach to analysis may have restricted the ability to explore unexpected findings and nuances and can increase the risk of confirmation bias. We acknowledge that while the evaluation was undertaken using approaches underpinned by the principles of PAR, it was not fully aligned with all aspects of PAR in its fullest sense due to the practicalities of the project and evaluation commission. While we managed to recruit a wide range participants, some of whom offered different perspectives, a limitation is that some of the project partners were not willing to take part in the evaluation. We therefore recognize that the findings may not reflect the views of everyone involved. We also reflect how, in a project which sought to engage and centre the experiences of minorities communities, both researchers were white and from academic institutions. This may have affected the relationships built with representatives from underrepresented communities and thus the interactions within, and the evaluation of, the project.

## Conclusions

This paper set out to understand the ways in which collaboration and co-production were used within a project working to address issues of maternal safety through the production of innovative co-produced content. The evaluation found that for effective collaborative co-production to take place, key issues relating to inclusivity and diversity as well as clarity and transparency about remit, commitments and finances needed to be addressed from the start of the project and throughout. The findings showed that it was crucial to ensure that time was spent in building and maintaining relationships between partners and communities within and beyond the project, and that this required a mix of face-to-face and virtual meetings. The creation of a safe space was essential to support partners to fully participate. An inclusive leadership style based on mutual respect and one which valued the knowledge and input of all involved acted to facilitate collaboration and cooperation. Findings also highlight how effective collaboration can provide benefits on an individual and organizational perspective, and that further work to ensure the sustainability of these relationships is needed. The learning from this project has implications for projects beyond midwifery in wider aspects of healthcare co-production and for projects seeking to work with diverse communities.

## Data Availability

No datasets were generated or analysed during the current study.

## References

[1] European Union Policy Department for Citizens’ Rights & Constitutional Affairs. Obstetric and gynaecological violence in the EU—Prevalence, legal frameworks & educational guidelines for prevention and elimination. 2014. https://www.europarl.europa.eu/RegData/etudes/STUD/2024/761478/IPOL_STU(2024)761478_EN.pdf. Accessed April 2024.

[2] Perrotte V, Chaudhary A, Goodman A. “At least your baby is healthy” obstetric violence or disrespect and abuse in childbirth occurrence worldwide: a literature review. Open J Obstetr Gynecol. 2020;10(11):1544–62.

[3] Pickles C, Herring J. Childbirth, vulnerability and law: exploring issues of violence and control. London: Routledge; 2019.

[4] Vedam S, Stoll K, Rubashkin N, Martin K, Miller-Vedam Z, Hayes-Klein H, Jolicoeur G. The mothers on respect (MOR) index: measuring quality, safety, and human rights in childbirth. SSM Popul Health. 2017;3:201–10. 29349217 10.1016/j.ssmph.2017.01.005 PMC5768993

[5] Fenech G, Thomson G. Tormented by ghosts from their past’: a meta-synthesis to explore the psychosocial implications of a traumatic birth on maternal well-being. Midwifery. 2014;30(2):185–93. 24411664 10.1016/j.midw.2013.12.004

[6] Acharya AK, Sarangi R, Behera SS. Experiences and impacts of obstetric violence on Indian women within the public healthcare system. J Feminist Gender Women Stud. 2021;11:37–45.

[7] Watson K, White C, Hall H, Hewitt A. Women’s experiences of birth trauma: a scoping review. Women Birth. 2021;34(5):417–24. 33020046 10.1016/j.wombi.2020.09.016

[8] Delicate A, Ayers S. The impact of birth trauma on the couple relationship and related support requirements; a framework analysis of parents’ perspectives. Midwifery. 2023;123: 103732. 10.1016/j.midw.2023.103732. 37229840 10.1016/j.midw.2023.103732

[9] Mayra K, Matthews Z, Sandall J. ‘Surrogate decision-making’ in India for women competent to consent and choose during childbirth. Agenda. 2021;35(3):92–103. 10.1080/10130950.2021.1958549.

[10] Silverio SA, Varman N, Barry Z, et al. Inside the ‘imperfect mosaic’: minority ethnic women’s qualitative experiences of race and ethnicity during pregnancy, childbirth, and maternity care in the United Kingdom. BMC Public Health. 2023;23:2555. 10.1186/s12889-023-17505-7. 38129856 10.1186/s12889-023-17505-7 PMC10734065

[11] OjiNjideka Hemphill N, Crooks N, Zhang W, Fitter F, Erbe K, Rutherford JN, Liese KL, Pearson P, Stewart K, Kessee N, Reed L, Tussing-Humphreys L, Koenig MD. Obstetric experiences of young black mothers: an intersectional perspective. Soc Sci Med. 2023;317: 115604. 10.1016/j.socscimed.2022.115604. 36549014 10.1016/j.socscimed.2022.115604 PMC9854070

[12] Hulley J, Bailey L, Kirkman G, Gibbs GR, Gomersall T, Latif A, Jones A. Intimate partner violence and barriers to help-seeking among black, asian, minority ethnic and immigrant women: a qualitative metasynthesis of global research. Trauma Viol Abuse. 2022;24(2):1001–15. 10.1177/15248380211050590. 10.1177/15248380211050590 PMC10012394 35107333

[13] Sambrook Smith M, Lawrence V, Sadler E, Easter A. Barriers to accessing mental health services for women with perinatal mental illness: systematic review and meta-synthesis of qualitative studies in the UK. BMJ Open. 2029;9(1):e024803. 10.1136/bmjopen-2018-024803. 10.1136/bmjopen-2018-024803 PMC6347898 30679296

[14] Messiha K, Chinapaw JM, Ket H, et al. Systematic review of contemporary theories used for co-creation, co-design and co-production in public health. J Public Health. 2023;45(3):723–37. 10.1093/pubmed/fdad046 PMC10470345 37147918

[15] Abma TA, Pittens CACM, Visse M, Elberse JE, Broerse JEW. Patient involvement in research programming and implementation: a responsive evaluation of the dialogue model for research agenda setting. Health Expect. 2015;18(6):2449–64. 10.1111/hex.12213. 24889933 10.1111/hex.12213 PMC5810623

[16] Brien O. An overview of the methodological approach of action research. Am Inst Res. 1998;2(4):1–14.

[17] Gillis A, Jackson W. Research methods for nurses: methods and interpretation. Davis Company. 2002.

[18] Haggar T, Boulding, H, Pow R, et al. Advancing co-production in local maternity services. Kings College, London. 2024. Accessed Feb 2025.

[19] Department of health and social care. Women’s health strategy for England. 2022. https://www.gov.uk/government/publications/womens-health-strategy-for-england/womens-health-strategy-for-england Accessed Feb 2025.

[20] Piggot-Irvine E, Rowe W, Ferkins L. Conceptualizing indicator domains for evaluating action research. Educ Action Res. 2015;23(4):545–66.

[21] Dedding C, Goedhart NS, Broerse JE, Abma TA. Exploring the boundaries of ‘good’ participatory action research in times of increasing popularity: dealing with constraints in local policy for digital inclusion. Educ Action Res. 2021;29(1):20–36.

[22] Patton MQ. Enhancing the quality and credibility of qualitative analysis. Health Serv Res. 1999;34(5):1189. 10591279 PMC1089059

[23] Braun V, Clarke V. Reflecting on reflexive thematic analysis. Qual Res Sport Exer Health. 2019;11(4):589–97.

[24] Albert A, Islam S, Haklay M, McEachan RRC. Nothing about us without us: a co-production strategy for communities, researchers and stakeholders to identify ways of improving health and reducing inequalities. Health Expect. 2023;26(2):836–46. 10.1111/hex.13709. 36683204 10.1111/hex.13709 PMC10010091

[25] Farr M, Davies P, Andrews H, et al. Co-producing knowledge in health and social care research: reflections on the challenges and ways to enable more equal relationships. Humanit Soc Sci Commun. 2021;8:105. 10.1057/s41599-021-00782-1.

[26] Grindell C, Coates E, Croot L, O’Cathain A. The use of co-production, co-design and co-creation to mobilise knowledge in the management of health conditions: a systematic review. BMC Health Serv Res. 2022;22(1):877. 10.1186/s12913-022-08079-y. 35799251 10.1186/s12913-022-08079-y PMC9264579

[27] National Institute for Health Research (NIHR). Guidance on coproducing a research project. 2021 https://www.learningforinvolvement.org.uk/content/resource/nihr-guidance-on-co-producing-a-research-project/. Accessed May 2024.

[28] CFE Research & The Systems Change Action Network. Coproduction Principles into practice Evidence from the Fulfilling Lives programme: Supporting people experiencing multiple disadvantage.2022. https://www.tnlcommunityfund.org.uk/media/insights/documents/Coproduction-Principles-into-practice-2022.pdf?mtime=20220715095830&focal=none. Accessed Apr 2024.

[29] Social Care Institute for Excellence (SCIE). Co-production: what it is and how to do it. 2022. https://www.scie.org.uk/co-production/what-how/. Accessed May 2024.

[30] NHS Providers. Co-production and engagement with communities as a solution to reducing health inequalities. 2024. https://nhsproviders.org/media/698572/co-production-health-ineq-1e.pdf. Accessed May 2024.

[31] Schroeder D. Benefit sharing: it’s time for a definition. J Med Ethics. 2007;33(4):205–9. 10.1136/jme.2006.016790. 17400617 10.1136/jme.2006.016790 PMC2652775

[32] Masterson D, Areskoug Josefsson K, Robert G, Nylander E, Kjellström S. Mapping definitions of co-production and co-design in health and social care: a systematic scoping review providing lessons for the future. Health Expect. 2022;25(3):902–13. 35322510 10.1111/hex.13470 PMC9122425

[33] Richards DP, Jordan I, Strain K, Press Z. Patient partner compensation in research and health care: the patient perspective on why and how. Patient Exper J. 2018;5(3):6–12. 10.35680/2372-0247.1334.

[34] Wechsler AM. Overcoming the Venn diagram: learning to be a co-passionate navigator in community-based participatory research. Res All. 2017. 10.18546/RFA.01.1.12.

[35] Knowles SE, Allen D, Donnelly A. et al. More than a method: trusting relationships, productive tensions, and two-way learning as mechanisms of authentic co-production. Res Involv Engagem. 2021;7. 10.1186/s40900-021-00262-5. 10.1186/s40900-021-00262-5 PMC8165763 34059159

[36] Amico L. The realities of remote work. The Harvard Business Review. 2021.

[37] https://hbr.org/2021/10/the-realities-of-remote-work. Accessed Apr 2024.

[38] Karl KA, Peluchette JV, Aghakhani N. Virtual work meetings during the COVID-19 pandemic: the good, bad, and ugly. Small Group Res. 2022;53(3):343–65. 10.1177/10464964211015286. 38603094 10.1177/10464964211015286 PMC8165498

[39] King C, Gillard S. Bringing together coproduction and community participatory research approaches: using first person reflective narrative to explore coproductionco-production and community involvement in mental health research. Health Expect. 2019;22(4):701–8. 10.1111/hex.12908. 31187556 10.1111/hex.12908 PMC6737774

